# Association of virulence gene expression with colistin-resistance in *Acinetobacter baumannii*: analysis of genotype, antimicrobial susceptibility, and biofilm formation

**DOI:** 10.1186/s12941-018-0277-6

**Published:** 2018-06-01

**Authors:** Abbas Bahador, Zahra Farshadzadeh, Reza Raoofian, Masoumeh Mokhtaran, Babak Pourakbari, Maryam Pourhajibagher, Farhad B. Hashemi

**Affiliations:** 1 Department of Microbiology, School of Medicine, Tehran University of Medical Sciences, 100 Poursina Ave., 100 Keshavarz Blvd, Tehran, 14167-53955 Iran; 2 Legal Medicine Research Center, Legal Medicine Organization, Tehran, Iran; 3 Innovative Research Center, Islamic Azad University, Mashhad Branch, Mashhad, Iran; 4 Pediatrics Infectious Disease Research Center, Tehran University of Medical Sciences, Tehran, Iran; 5 Dental Research Center, Dentistry Research Institute, Tehran University of Medical Sciences, Tehran, Iran; 6 Laser Research Center, Dentistry Research Institute, Tehran University of Medical Sciences, Tehran, Iran

**Keywords:** *Acinetobacter baumannii*, Colistin resistance, Virulence gene expression, Murine burn model

## Abstract

**Background:**

*Acinetobacter baumannii* causes difficult-to-treat nosocomial infections, which often lead to morbidity due to the development of antimicrobial drug resistance and expression of virulence genes. Data regarding the association of resistance to colistin, a last treatment option, and the virulence gene expression of *A. baumannii* is scarce.

**Methods:**

We evaluated the MLVA genotype, antimicrobial resistance, and biofilm formation of 100 *A. baumannii* isolates from burn patients, and further compared the in vitro and in vivo expression of four virulence genes among five colistin-resistant *A. baumannii* (Cst-R-AB) isolates. Five Cst-R-AB isolates were tested; one from the present study, and four isolated previously.

**Results:**

Our results showed that reduced expression of *recA*, along with increased in vivo expression of *lpsB*, *dnaK*, and *blsA*; are associated with colistin resistance among Cst-R-AB isolates. Differences in virulence gene expressions among Cst-R-AB isolates, may in part explain common discrepant in vitro vs. in vivo susceptibility data during treatment of infections caused by Cst-R-AB.

**Conclusions:**

Our findings highlight the intricate relationship between colistin-resistance and virulence among *A. baumannii* isolates, and underscore the importance of examining the interactions between virulence and antimicrobial resistance toward efforts to control the spread of multidrug-resistant *A. baumannii* (MDR-AB) isolates, and also to reduce disease severity in burn patients with MDR-AB infection.

**Electronic supplementary material:**

The online version of this article (10.1186/s12941-018-0277-6) contains supplementary material, which is available to authorized users.

## Background

*Acinetobacter baumannii* is an opportunistic pathogen that can cause formidable infections among patients with burn wounds worldwide [1]. Also, the World Health Organization (WHO) has announced *A. baumannii* as “Critical” priority pathogenic bacteria that pose the greatest threat to human health. Treatment of *A. baumannii* wound infection is often difficult; primarily due to the pathogenic factors, which enable the establishment of persistent infections within burn patients leading to high morbidity and mortality [2–4]. These pathogenic factors mainly include the development of multi-drug resistance, and production of virulence determinants, such as biofilm formation [5–7]. Reports from various parts of the world have indicated a worrisome growing trend of isolating multi-, extensive- and pan-drug resistant (MDR, XDR, and PDR) strains of *A. baumannii,* some of which are even resistant to colistin, a last resort drug [8–13]. Whether the expression of virulence determinants, such as genes involved in biofilm formation, play an important role in the high rate of treatment failure among wound infections caused by highly resistant *A. baumannii* isolates remains to be explored [14].

To date, the expression of several *A. baumannii* virulence genes has been linked to the persistence and enhanced survival of *A. baumannii* within the host, including quorum sensing genes [15], and genes that confer biofilm production, which ultimately lead to increased antimicrobial resistance [16, 17]. Furthermore, the activation of *A. baumannii* virulence determinant genes, such as *recA* and *dnaK,* can render robust strains, which are less vulnerable to host response stresses [18]. Moreover, the expression of *recA* by *A. baumannii* isolates increases resistance to stress [19], while *recA* inactivation increases susceptibility to a variety of antimicrobial agents [15, 20].

Then again, mounting evidence suggests that increased virulence gives rise to isolates that are less “fit” to survive in their host, and renders them more susceptible to antimicrobial agents [21]. For instance, the loss of glycosyl transferase gene (*lps*B)], essential for *A. baumannii* lipopolysaccharide (LPS) core biosynthesis, corresponds to the phenotypes of “reduced survival rate”, attenuated biofilm formation, and increased antimicrobial susceptibility [22–24]. Similarly, the expression of blue light sensing (*blsA*) gene and production of BlsA protein has been demonstrated to inhibit biofilm formation, which also leads to increased susceptibility to antimicrobial agents [25, 26]. In addition, among *A. baumannii* clinical isolates, the development of colistin resistance (Cst^R^) has been shown to enhance biofilm formation, but reduce their invasiveness [27, 28]. Although these reports present evidence of likely interactions between virulence and the antimicrobial susceptibility profile of *A. baumannii*, the association of the expression of specific virulence genes with antimicrobial susceptibility has not been thoroughly investigated. Likewise, data comparing the expression of virulence genes among colistin-resistant *A. baumannii* (Cst-R-AB) strains is scarce.

In the present study, we aim to examine whether resistance to colistin in *A. baumannii* isolates is associated with the expression of specific virulence genes. We also analyze the genotypes of the *A. baumannii* isolates, their biofilm formation ability, and antimicrobial susceptibility profiles. By revealing the interaction of virulence genes with Cst^R^ among *A. baumannii* isolates, we attempt to explain, at least in part, the apparent discrepancies between in vitro antimicrobial resistance data, and in vivo clinical outcomes in *A. baumannii* infected burn patients. Our findings may ultimately help devise strategies toward effective treatments of *A. baumannii* burn wound infections, as well as make prudent decisions regarding other infection control measures to thwart nosocomial outbreaks of MDR *A. baumannii*.

## Methods

### Specimens, bacterial isolates and cultures

One hundred non-repetitive clinical specimens were collected from the burn unit of the Burn Medical Center Complex in Tehran, Iran; during April–December 2014. After admission, written informed consent forms were obtained from either the patients, or their authorized representatives. This study was approved by Tehran University of Medical Sciences (TUMS) Research Ethics Committee (Application No. TREC-89. 01-30-10430). All 100 burn wound isolates were initially identified as *A. baumannii* using the API20NE system (bioMérieux, Marcy-l’Etoile, France), and later confirmed using the *gyrB* multiplex PCR, as described previously [29]. Mueller–Hinton Broth (MHB) and brain heart infusion (BHI) agar plates (both from Merck, Darmstadt, Germany) were used to culture the bacterial isolates under aerobic conditions for 24 h at 37 °C. In total, five Cst-R-AB isolates were analyzed including, a single Cst-R-AB isolates from this study, and four additional Cst-R-AB isolates from previous cohort studies.

### Antimicrobial susceptibility testing

To assess antimicrobial susceptibility patterns of *A. baumannii* clinical isolates, we carried out the disk agar diffusion (DAD) method according to the Clinical and Laboratory Standards Institute (CLSI) procedures [30] and breakpoint interpretations, using antimicrobial disks containing the 17 antimicrobial agents: ampicillin–sulbactam, cefepime, ceftazidime, ciprofloxacin, colistin, gentamicin, imipenem, levofloxacin, meropenem, minocycline, piperacillin, piperacillin-tazobactam, rifampicin, tetracycline, tigecycline, tobramycin, and trimethoprim-sulfamethoxazole (Mast Diagnostics, Bootle, UK). The CLSI guideline for Broth microdilution test for minimum inhibitory concentration (MIC) was used to assess isolate susceptibility to colistin, rifampicin, and tigecycline. Colistin MICs were interpreted according to the CLSI breakpoints [30]. For tigecycline susceptibility tests, the criteria of the European Committee on Antimicrobial Susceptibility Testing (EUCAST) [31] for *Enterobacteriaceae* were used, in which an MIC of < 1 µg/mL was defined as susceptible and > 2 µg/mL was considered resistant [31]. Rifampicin susceptibility was interpreted according to CLSI criteria using breakpoint values suggested for *Staphylococcus aureus*, in which susceptible and resistant were defined as ≤ 1 and ≥ 4 µg/mL, respectively [30]. *A. baumannii* isolates were defined as multi-, extensive- and pan-drug resistant (MDR, XDR, and PDR) according to the definitions described previously [32]. Antimicrobial agents were categorized into three groups; namely, Group A that were agents deemed appropriate for primary testing panel; Group B agents, for which primary testing may be warranted, but they may be reported selectively (such as when the organism is resistant to agents of the same class, as in group A). Also, Group O (other) that comprised agents that had a clinical indication for *A. baumannii*, but were not candidates for primary testing panel in the USA [30]. Colistin resistance (Cst^R^) among *A. baumannii* isolates was confirmed by the genetic analysis of the *pmr* operon showing mutations in *pmrA/pmrB* signal transduction system, which confers Cst^R^ by modifying lipid A phosphoethanolamine moiety of the lipopolysaccharide (LPS) layer in the outer membrane.

### International clones, MLVA genotype analysis

International clone (IC) types of the isolates were determined based on the presence of *ompA*, *csu*E, and *bla*_OXA-51_-like allele amplicons using two complementary multiplex PCR assays, as previously described [33]. Table 1 shows the primers used in the multiplex PCR assays, which selectively amplified the outer membrane protein A (ompA), chaperone-subunit usher E (csuE), and *bla*_OXA-51-like_ intrinsic carbapenemase genes of the *A. baumannii* isolates. Isolates not assigned as either IC type I, II, or type III were reported as the variant clonal type (IC-V).

**Table 1:** Primer sequences used in this study

Primer name	Sequence (5′–3′)	Amplicon size (bp)
*blsA*-F	ACCTTTAACCCGCTTTTGCT	117
*blsA*-R	TCCCCTATTCACCATTCCAA	
*lpsB*-F	AGGCCATCAATCTTTGGTTG	137
*lpsB*-R	GCTGACGTAATGGACGGATT	
*dnaK*-F	GCGTTTAATTGGTCGTCGTT	105
*dnaK*-R	ACTTCAACCCAAGCATCACC	
*recA*-F	CACGCCCTAGACCCTCAATA	136
*recA*-R	CGATTAAATCAATTGCGCCT	
*16srRNA*-F	AAAGTTGGTATTCGCAACGG	117
*16srRNA-*R	ACCTTTAACCCGCTTTTGCT	

To examine the genotypic diversity of *A. baumannii* clinical isolates, multi-loci variable-number tandem repeat analysis (MLVA) was carried out, as previously described [34]. Briefly, DNA from the *A. baumannii* isolates was extracted using a GeneJET DNA purification kit and its concentration was assessed using a NanoDrop 1000 spectrophotometer (Thermo Scientific, USA). Genomic DNA (25 ng/µL) was assayed by PCR using a 30 μL final volume containing 5 ng of DNA, 10× reaction buffer (SinaClon BioScience, Iran), 1.5 mM MgCl [1, 2] U of Taq DNA polymerase, 200 μM each deoxynucleotides (dNTP), and 0.3 μM of each primer (all provided by SinaClon BioScience, Iran). Amplification cycles included an initial 94 °C denaturation for 5 min, followed by 35 cycles of 94 °C for 30, 30 s of annealing at 50 or 55 °C depend on melting temperature of the primers, elongation at 72 °C for 30 s, and a final elongation at 72 °C for 10 min. Then, 5 μL of each PCR amplicon solution were analyzed on a 25 cm 3% agarose gel (SinaClon BioScience, Iran) by electrophoresis (3 h at 6 V/cm) in 1× Tris–borate-EDTA (TBE) buffer. Size markers included a 100-bp (SinaClon Bioscience), and 50-bp DNA standards (Thermo Scientific) for L- and S-variable number tandem repeats (VNTRs), respectively. The amplicon bands were visualized with UV illumination after ethidium bromide (0.5 μg/mL) staining. Band sizes were determined using GeneTools v.3.08 automatic image analysis software (Syngene, Cambridge, United Kingdom). For cluster analysis, allele strings were analyzed by BioNumerics software v.7.0 as character values (Applied Maths, Sint-Martens-Latem, Belgium). Clusters were defined using cut-off values of 95% similarity, and MLVA type was determined using a 100% similarity cutoff, as previously described [35].

### Semi-quantitative biofilm formation assay

The semi-quantitative assessment of biofilm formation was performed in triplicate using crystal violet staining, as previously described [36]. Briefly, *A. baumannii* isolates were cultured on BHI agar overnight, and an isolated colony was suspended in LB Broth (Himedia, India) for 4 h. Bacterial suspensions (200 µL) in logarithmic growth phase were adjusted to 0.5 McFarland’s standard [1.5 × 10^8^ colony forming units (CFU)/mL] and incubated at 35 °C in flat-bottomed 96-well plates. After 48 h, media was removed, and wells were washed three times with dH_2_O. Then, 200 µL of 1% crystal violet dye was added and plates were incubated at room temperature (RT) for 20 min. The dye was then decanted, wells were washed twice with dH_2_O and dried. Finally, 200 µL of 95% ethanol (200 µL/well) was added, and the optical absorbance (A) was measured at 570 nm (Thermo Scientific GmbH, Driesch, Germany). Reference strain (ATCC DH5α) was used as negative control (NC) to assign scores for biofilm formation, according to following formula: negative isolate (N) = A_I_ ≤ A_NC_; weakly positive isolate (W) = A_NC_ < A_I_ ≤ 2A_NC_; moderately positive isolate (M) = 2A_NC_ < A_I_ ≤ 4A_NC_; and strongly positive isolate (S) = A_I_ > 4A_NC_.

### Virulence genes detection

To detect virulence factor genes *dnaK*, *recA*, *lpsB* and *blsA*, a series of PCR assays were utilized using primers (Table 1; Primer 3 software v.4.0). Briefly, 1 μL of genomic DNA (25 ng) from Cst-R-AB strains was assessed by PCR using 12.5 μL of 2× PCR Master Mix, and 1 μL (2.5 pM) of each primer (both from SinaClon BioScience, Iran). Amplifications were performed using a Mastercycler Personal (Eppendorf^®^, Germany), with initial denaturation for 5 min at 94 °C, and 35 cycles at 94 °C for 30 s, annealing for 30 s at 52 °C (Table 1), followed by elongation at 72 °C for 30 s, and a final step at 72 °C for 5 min. The presence of gene specific amplicons was verified visually by 2% agarose gel electrophoresis using ethidium bromide staining.

### Animal study design

All animal experiments were carried out in accordance to the protocols approved by the Animal Ethics Committee of Tehran University of Medical Sciences (Application No. TUMS-AEC-89-0130-10430). Male C57BL/6 mice (6–8 week old; 18–23 g; Pasteur Institute, Tehran, Iran) were housed one mouse per cage, under sanitary conditions at 22–25 °C and at 12 h light/dark cycles, with access to sanitized pellet food and water. Mice were acclimated to room conditions for 1 week prior to each experiment. To increase the accuracy of microbiological assessments, the cages were disinfected with povidone iodine solution 10%, and the bedding materials were autoclaved, and replaced every day.

Based on mortality rates of burn wound infection from previous studies, the effect size (No. of mice in experimental groups) was estimated as five mice per each isolate, using power analysis with power arbitrarily set at 80%. Initially, two groups (five mice per group) were infected with the only Cst-R-AB (MT10) isolated in this study. One group was infected with colistin-treated Cst-R-AB isolate, and the other with Cst-R-AB isolate with no colistin treatment. Mock-infected burned mice (C-Burn) served as controls, and received physiological saline instead of *A. baumannii* infection (third group).

Similarly, groups of mice were later infected with additional four Cst-R-AB isolates (MIC 32–256 µg/mL), recovered from burn wounds in a previous study [37]. In order to expose the Cst-R-AB isolates to colistin, and evaluate the putative colistin-induced changes in virulence gene expression, the group of colistin-treated Cst-R-AB infected mice were administered with colistin (20 µg/g weight); which yielded sub-MIC serum levels of approximately 25 µg/µL [38]. For analysis of all five (i.e. 1 + 4) Cst-R-AB isolates, all in vivo data were combined and presented as Mean + SD.

### Murine burn wound infection and bacterial quantification

Burn wounds were generated, as described previously [39]. Briefly, mice were anesthetized by an intraperitoneal (i.p.) injection of a ketamine–xylazine cocktail, after which the dorsal/posterior surface was shaved, and each mouse was then injected with 0.5 mL (i.p.) of sterile saline to prevent dehydration. A brass 10 mm × 10 mm block preheated to 95 °C was applied to the shaved area for a 10 s period. This procedure consistently generated a nonlethal, full-thickness, third-degree burn. Burn wound infections were established as previously described [40]. Briefly, 5 min after inducing the burn wound, using a sterile inoculating loop, a 50 µL suspension of each Cst-R-AB isolate containing 1.1–2.1 × 10^5^ CFU/mL was smeared onto the burn wound site. After 72 h, bacterial concentrations in wound sites of mice infected with un-treated Cst-R-AB were compared to mice infected with Cst-treated Cst-R-AB isolate (treated with sub-optimal i.e. < MIC). Viable bacteria quantitated by colony enumeration of bacteria harvests from the wound bed, and reported as CFU/mL by using biopsy punch method as previously described [41]. All mice were euthanized via an overdose of ketamine (250 mg/kg) and xylazine (25 mg/kg) prior to bacterial harvest from the wound site.

### RNA extraction and cDNA synthesis and in vivo virulence gene expression

To measure in vivo virulence genes expression within the wound bed skin, tissue specimens were collected from the infected tissue from each sacrificed mouse were processed separately. Briefly, wound bed skin tissue specimens were aseptically excised (2 mm deep), placed in PBS, and frozen in liquid N_2_ for immediate RNA extraction.

To compare the relative quantities of gene-specific mRNA of Cst-R-AB isolates under in vivo vs. in vitro conditions RNA extraction was carried out. For the “in vivo samples” mRNA was extracted from Cst-R-AB isolates in their respective burn wound sites, 72 h after inoculation. The in vitro mRNA samples were, however, prepared using Cst-R-AB isolates grown in culture media, as described above. In all comparative analyses, 16S rRNA gene mRNA served as control to normalize mRNA quantities among the samples. Total bacterial RNA of planktonic mid-log phase cultures of Cst-R-AB isolates were extracted using the Total RNA Extraction Kit (iNtRON Biotech Inc., Seoul, South Korea). To extract bacterial RNA from tissue, wound specimens were ground and homogenized in liquid nitrogen, and the cellular lysates were run through All Prep DNA/RNA columns. Total RNA extraction was performed using RNeasy Plus Mini Kit (QIAGEN, Hilden, Germany) following the manufacturer’s recommendations. Bacterial mRNA enrichment was achieved by step-wise treatment of total RNA samples to eliminate mouse mRNA [NEBNext Poly(A) mRNA Magnetic Isolation Module, New England Biolabs, USA], followed by the removal of mouse and bacterial rRNA (Ribo-Zero Gold rRNA Removal Kit, Epicentre Biotechnologies, USA). RNA purity was determined by 260/280 and 260/230 nm absorbance ratio using a NanoDrop 1000 spectrophotometer (Thermo Scientific, USA), and size-verified by 2% agarose gel electrophoresis prior to RT-PCR and qRT-PCR assays. Before generating cDNA, bacterial mRNA samples were treated with DNase I (Thermo Scientific, USA) and RevertAid First Strand cDNA Synthesis Kit (Thermo Scientific, USA). Samples of 1 μg total RNA were used to generate cDNA libraries, for the reverse transcription (RT)-PCR, and quantitative real time RT-PCR (qRT-PCR) assays.

### Reverse transcriptase (RT-PCR) and quantitative (qRT)-PCR assays

RT-PCR assay was used to assess the expression of *dnaK, blsA, recA,* and *lpsB* genes. Briefly, first-strand cDNA served as a template for the amplification of virulence genes, and qRT-PCR was used to measure the relative changes in mRNA levels as indicators of virulence gene expression. Samples of cDNA from Cst-R-AB isolates were analyzed by qRT-PCR assay using the primer sets shown in Table 1. The Line-Gene K Real Time PCR System (BIOER Technology Co., China) was used to perform the qRT-PCR according to the minimum information for publication of quantitative real-time PCR experiments (MIQE) guidelines [42] under the following conditions: 95 °C for 15 min, followed by 45 cycles of 95 °C for 15 s, annealing for 30 s at 60, and 72 °C for 30 s, using 10 µL of SYBR^®^ Premix Ex Taq™ II (Tli RNaseH Plus) (TaKaRa Bio Inc., Japan), 2 µL of gene-specific forward and reverse primers (2.5 pmol), cDNA template (50 ng), and 7 µL of ddH_2_O (total 20 µL). The quantity of gene-specific amplicons was determined by qPCR relative to an internal standard (16S rRNA) used as a calibrator. Target amplicons were checked for size and specificity by agarose gel electrophoresis and melting curve analysis, respectively. The changes in the expression level of each virulence gene was calculated by the 2^−∆∆Ct^ method using the Relative Expression Software Tool (REST) 2009 software (version 2.0.13; Qiagen, Valencia, CA, USA) [44]. Difference of > 4-fold relative to basal gene expression levels was considered significant, and referred to as *n*-fold difference.

### Statistical analysis

Data were analyzed using the Student’s t-test, and the Chi square test using the SPSS software package (version 22). Results were considered significant if *P* < 0.05.

## Results

### Antimicrobial susceptibility profiles

**Fig. 1:**
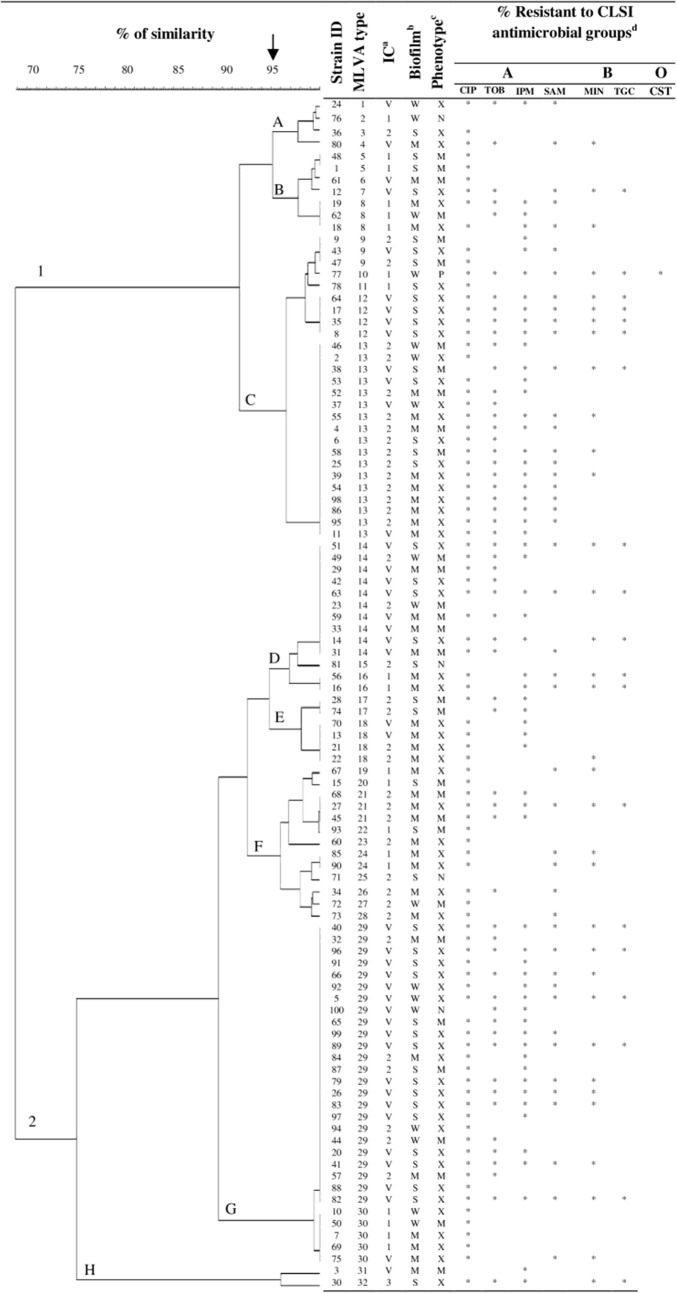
Comparison of MLVA genotype diversity of 100 *Acinetobacter baumannii* isolates (dendogram) with their biofilm formation phenotype, as well as resistance to CLSI antimicrobial groups, and international clonal lineage

As shown in Fig. 1, the majority (95%) of *A. baumannii* isolates had a MDR (31%) or XDR (64%) profile of antimicrobial resistance, while the frequency of non-MDR, or PDR isolates was 4%, and 1%, respectively. While most isolates (96%) were resistant to at least three classes of antimicrobial agents, nearly 90% were resistant to at least 10 tested antimicrobial agents including; cephalosporins, trimethoprim/sulfamethoxazole, tetracycline, piperacillin/tazobactam, and fluoroquinolones. Figure 1 also shows the susceptibility profile of *A. baumannii* isolates against seven antimicrobial agents representing the A, B, and O groups of antimicrobial agents [30]. The highest resistance rates were among group A (45–96%), followed by group B (19–97%), and group O antimicrobial agents (1%). The lowest rates of resistance were against colistin (1%), tigecycline (19%), and minocycline (42%). Moreover, resistance rates to ciprofloxacin, tobramycin, and imipenem were 89, 64 and, 60% respectively. Surprisingly, the rate of resistance to tobramycin was higher among MDR isolates (82%) than the XDR (87%) isolates (*P* > 0.05).

### MLVA genotyping and international clone analysis

International clone (IC) type analysis showed that the majority of *A. baumannii* isolates were IC-V variants (43%), followed by IC-II (37%), IC-I (19%), and IC-III types (1%). Also, MLVA genotype analysis (95% allelic similarity) revealed eight different strain clusters, including strain clusters A, B, and C in one group, as well as clusters D through H in another group (Fig. 1).

Overall, we identified 32 MLVA types (MT), which consisted of 1–24 members each (at 100% similarity cutoff). While most MTs had a single member, the three main MTs were MT29 (n = 24), MT13 (n = 17), and MT14 (n = 10), which together comprised over 50% of all isolates (Fig. 1). The majority of MT29 (18/24) and MT14 (8/10) members were IC-V variants, which were predominantly XDR isolates. Specifically, 83% (15/18) of MT29 IC-V variants, and 50% (4/8) of MT14 variants showed XDR profiles. The small MT12 cluster (n = 4) was remarkable since all four were IC-V and XDR isolates. Approximately 75% of the MT29 isolates and 40% of the MT14 isolates were categorized as IC-V. Among the MT29, MT13, and MT14 isolates, XDR isolates predominated and showed similar frequencies. Most MT13 isolates (76%) were IC-II types with nearly 70% of these being XDR strains (Fig. 1).

### Association of MDR profile with IC types and biofilm formation index

**Fig. 2:**
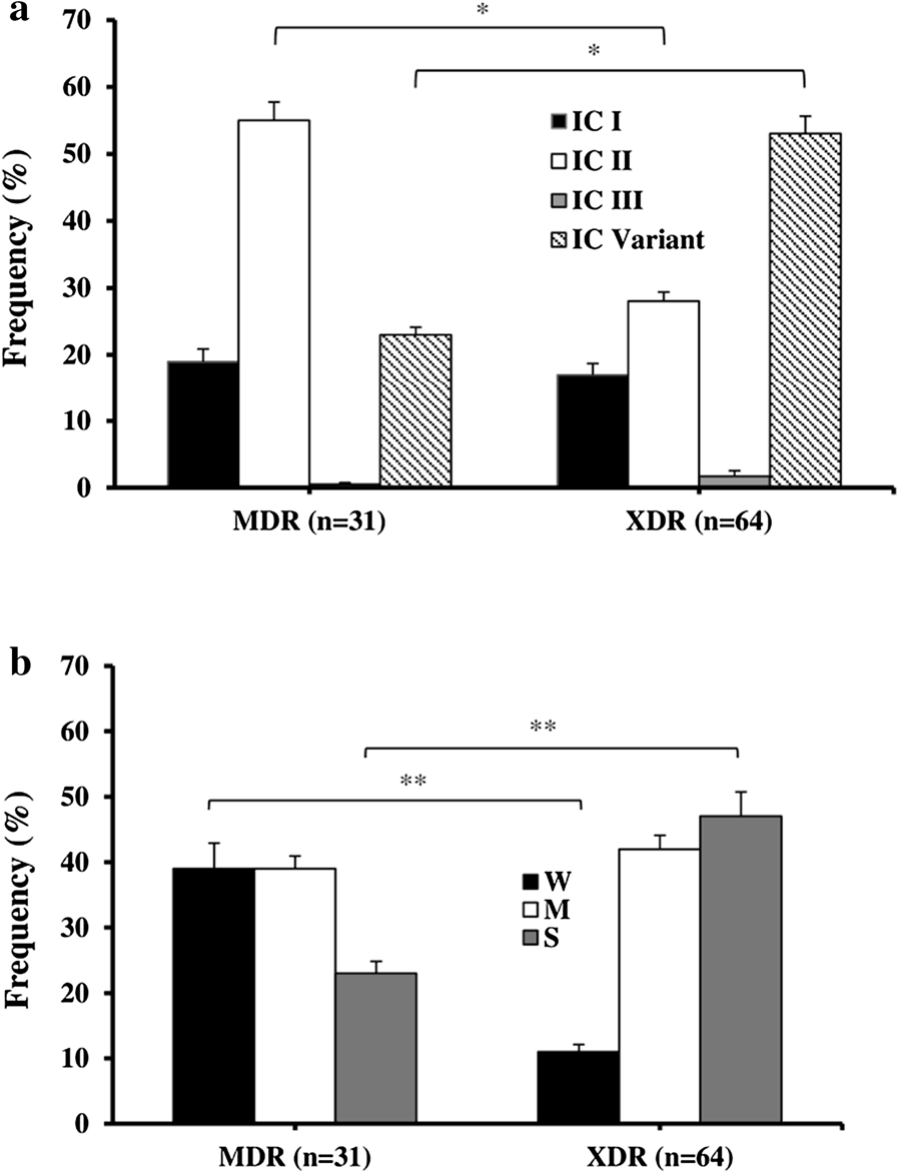
Comparison of MDR and XDR isolates’ frequency according to their IC type **a**; and, the frequency of MDR and XDR isolates with weak (W), moderate (M), and strong (S) biofilm formation **b**. Bars indicate Mean + SEM; *P < 0.05; **P < 0.01, between the indicated pairs

We further examined the association of the MDR profile of antimicrobial resistance of each isolate with the ability to form biofilm as a virulence determinant, as well as the IC type of each isolate (Fig. 2a, b). Our analysis focused on comparing the biofilm formation index of each isolate with the isolate MDR and XDR profile, since 95% of all isolates had either an MDR or XDR profile. Figure 2a shows that the majority of XDR isolates were IC variants, whereas MDR isolates were predominantly IC-II type. The frequency of IC-II isolates with MDR profile (55%) was almost two folds higher than that of those with XDR profiles (28%; *P* = 0.031). In contrast, the frequency of IC-V isolates with MDR profiles was nearly half the frequency of those with XDR profiles (23% vs. 53%; *P* = 0.021). The frequencies of MDR vs. XDR profiles among IC-I and IC-III isolates was similar (Fig. 2a).

Comparison of the antimicrobial resistance profile of the isolates’ with their biofilm strength index revealed that 77% of the W- and M-biofilm forming isolates had either an MDR, or XDR profile. While, the S-biofilm formers comprised only 23% of MDR isolates, the single PDR isolate was a W-biofilm former. All MT12 members (n = 4) were IC-V type and S-biofilm formers that showed an XDR antimicrobial susceptibility profile. Among XDR isolates, the frequency of S- and M-biofilm formers were 48 and 43%, respectively; while among the MDR isolates, W- and M-biofilm formers were isolated at an identical 39% rate (Fig. 2b). In contrast, the frequency of W-biofilm formers among MDR isolates was 39%, whereas only 12% of XDR isolates were W-biofilm formers (*P* = 0.009). Similarly, the frequency of S-biofilm forming isolates with the XDR, or MDR antimicrobial susceptibility profile was 47 and 23%, respectively (*P* = 0.004). Finally, all four non-MDR isolates were either W- or S-biofilm formers (50% frequency of isolation); i.e. they included no M-biofilm formers.

### Association of IC type with biofilm formation

**Fig. 3:**
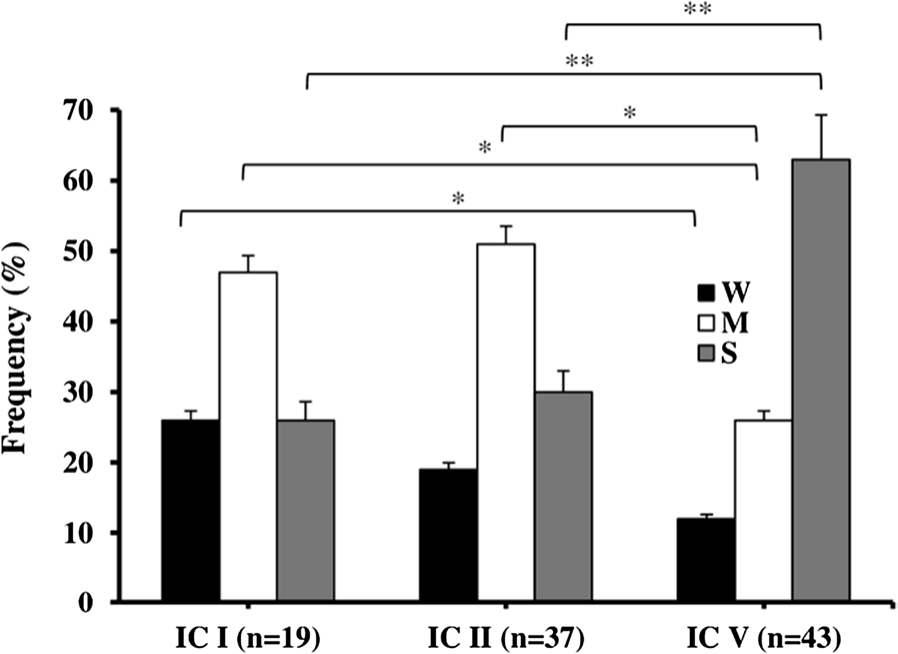
Frequency comparison of weak (W), moderate (M), and strong (S) biofilm forming *A. baumannii* isolates according to their IC lineage. Analysis included semi-quantitative measurements of biofilm formation strength (see “Methods” section). Bars indicate Mean + SEM; *P < 0.05; **P < 0.01

Additionally, we assessed whether the strength of biofilm formation was associated with the IC type of *A. baumannii* isolates. Figure 3 shows the frequency of W-, M- and S-biofilm formers among IC-I, IC-II, and IC-V isolates, which comprised 99% of all isolates. Among IC-V isolates, the frequency of S-biofilm formers (63%) was six folds higher than the W-biofilm formers (12%; *P* = 0.001); while, among IC-I and IC-II isolates, the frequencies of W-, M-, and S-biofilm formers were comparable, showing only 4–7% variation: i.e. 26% vs. 19% for W-biofilm formers (*P* = 0.382), and 26% vs. 30% for S-biofilm formers (*P* = 0.521), and finally 47% vs. 51% for M-biofilm formers (*P* = 0.437), in that order. As shown in Fig. 3, M-biofilm-forming isolates were almost twice as often seen among the IC-I and IC-II isolates compared to the IC-V isolates [47% vs. 26% for IC-I (*P* = 0.036), and 51% vs. 26% for IC-II (*P* = 0.028), respectively.

### Quantification of CFU concentration in wound bed tissues

There was no significant difference between the log CFU concentrations in the wound sites infected with un-treated Cst-R-AB isolate (7.78 ± 6.0), versus the log CFU concentrations (6.99 ± 5.82; P > 0.05) in wound beds that were infected with Cst-R-AB, which was treated with sub-MIC colistin. Thus, Cst-R-AB isolate concentrations (i.e. growth) were unaffected by the Cst-treatment of wound bed infection site.

### Virulence genes detection and expression in vitro and in vivo

**Fig. 4:**
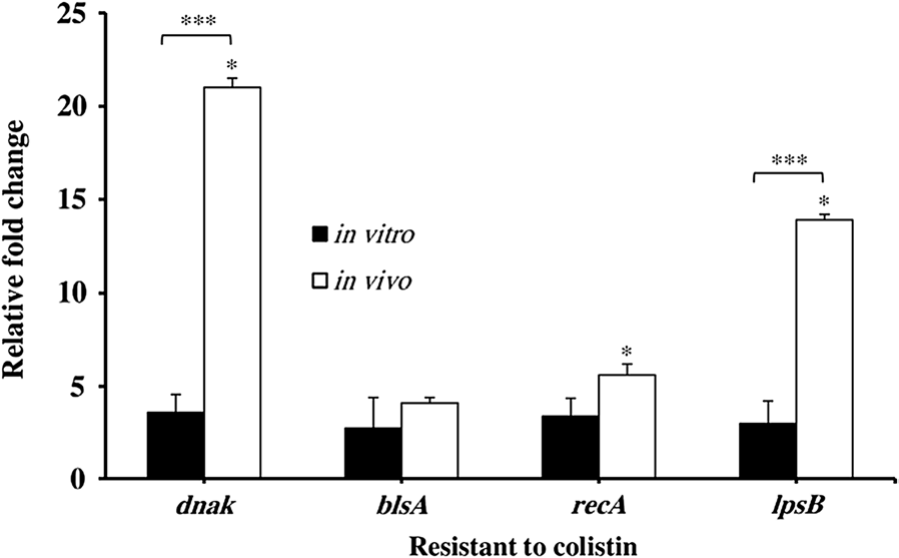
Comparison of relative change in the mRNA levels of virulence determinant genes *dnaK, blsA, recA,* and *lpsB* in cultures of five Cst-R-AB isolates, under in vitro and in vivo conditions. Bar indicates mean ± SD; five mice in each group. ^#^P < 0.01; *P < 0.05; ***P < 0.001

Virulence factor genes *dnaK*, *recA*, *lpsB* and *blsA*, were detected in the Cst-R-AB strain by PCR. In order to examine whether the expression of virulence genes *dnaK, blsA, recA,* and *lpsB* is associated with Cst resistance among isolates, we compared the relative gene-specific mRNA quantities of Cst-R-AB isolates under in vivo, vs. in vitro conditions (Fig. 4). The final data analysis included five Cst-R-AB isolates; including a single Cst-R-AB isolate from the present study (Additional file 1: Figure S1), and four additional Cst-R-AB isolates from previous studies.

Overall, as shown in Fig. 4, the expression levels of *dnaK, recA,* and *lpsB* genes in Cst-R-AB isolates were markedly different between in vivo vs. in vitro conditions. The relative change over basal expression levels of the four virulence genes, in vivo vs. in vitro, among Cst-R-AB isolates was reported as the Mean ± SD (Fig. 4).

Among the Cst-R-AB isolates, the in vivo expression of *dnaK* and *lpsB* was 23 and 14 folds higher than basal; respectively (Fig. 4); while *recA* mRNA levels showed a slight 3-fold increase (*P* < 0.05). Conversely, Cst-R-AB in vitro cultures revealed no significant increase in the expression of *dnaK, blsA,* and *lpsB*, with only a modest 4-fold rise in *recA* expression. Interestingly, under either condition, *blsA* gene mRNA levels of the Cst-R-AB isolates was up-regulated by 3–4-fold.

## Discussion

The emergence of multidrug-resistant *A. baumannii* (MDR-AB), as a major nosocomial pathogen among patients with burn wound infections, has drawn clinical attention to an old antimicrobial agent that is active against MDR-AB, namely colistin (Cst) [43, 44]. Unfortunately, reports from several regions of the world, including Iran, indicate that the widespread use of Cst, as a last resort to control MDR-AB infections, has led to a worrisome growing trend of Cst-resistance (Cst^R^) among MDR-AB strains [8, 45, 46]. Recurrently, the eradication of MDR-AB burn wound infections prove very challenging because, not only do clinical strains often develop antimicrobial resistance, but these strains also produce virulence factors (e.g. biofilm) that transform these MDR-AB into even more formidable wound pathogens [10, 12].

While, some studies have associated the presence of specific virulence genes with an increase in the pathogenic potential of clinical isolates [47, 48], the association between the virulence gene expression of *A. baumannii* and the susceptibility to specific antimicrobial agents has not been assessed. In fact, despite several reports regarding the increase in the prevalence of Cst-resistance among MDR-AB isolates worldwide [8–14, 45], few studies have focused on the possible link between Cst^R^, and the expression of virulence genes that can potentially affect therapy outcome. We have used a murine burn infection model to demonstrate that mRNA levels of virulence genes *dnaK, blsA, recA,* and *lpsB* vary in Cst-R-AB isolates depending on whether they are cultured in vitro or in vivo. Moreover, to examine the genotypic diversity among *A. baumannii* isolates, we have characterized their MLVA genotypes, IC lineage. We further evaluated any associations between the isolates’ IC lineage, susceptibility patterns, and their biofilm formation profiles.

Here, we also present evidence suggesting that the XDR or MDR profile of *A. baumannii* is associated with the biofilm strength. As shown in Fig. 2b, the frequency of W-biofilm formers among MDR isolates was about three times higher than among XDR isolates, which suggests that the strength of biofilm formation is related to the extended spectrum of antimicrobial resistance among isolates. Conversely, W- and M-biofilm formers were equally common (39%) among MDR isolates. That the XDR isolates had more M- and S-biofilm formers than MDR isolates suggests that the antimicrobial susceptibility profile is associated with biofilm strength among these isolates. However, since the rates of M-biofilm formation were similar among XDR and MDR isolates, the M-biofilm formation phenotype does not seem to follow this association pattern.

Interestingly, the frequency of S- and M-biofilm formers among XDR isolates were similar (48% vs. 43%, respectively); whereas among the MDR isolates, the W- and M-biofilm formers were isolated at an identical 39% rate (Fig. 2b). Conversely, the frequency of W-biofilm formers among MDR isolates were about three times higher than XDR isolates (*P* = 0.009), which suggests that strength of biofilm formation is associated with the isolate antimicrobial profile. Likewise, isolates with XDR antimicrobial susceptibility profiles were more than twice as likely to be S-biofilm formers, as compared to the frequency of S-formers among MDR isolates (*P* = 0.004). This suggests that stronger biofilm formation may be associated with broader antimicrobial resistance. The remarkable finding that all MT12 members were S-biofilm formers, and shared the same IC-V type and antimicrobial susceptibility profile is suggestive of a common source of isolation.

Interestingly, while IC-V variants had the highest frequency of S-biofilm formers, they included lowest frequency of W-biofilm formers. In addition, our data revealed that most of S-biofilm forming IC-V isolates also had XDR profiles, which is contrary to the reports that indicate biofilm formation is a common attribute of clinical *A. baumannii* isolates, regardless of their IC minor or major clonal variation [49–52]. This apparent discrepancy might be due to the unique features of our local IC-V isolates that have neither been isolated, nor characterized elsewhere. Consequently, the conclusion of studies that have reported no correlation between *A. baumannii* biofilm formation and IC lineage may be limited to the IC-I, -II, and -III categories, which apparently do not encompass a global representation of all *A. baumannii* isolates.

Here, we present evidence suggesting that the development of Cst^R^ may also lead to changes in *A. baumannii* virulence determinant genes expression, which in turn might enhance the ability to establish, and persist in burn wound infections. In order to further strengthen the scope of our initial findings, which were based on a single Cst-R-AB isolate, we expanded our in vivo experimental data by using four additional Cst-R-AB isolates in a similar murine model (Fig. 4). Since Cst^R^ has been shown to cause increased outer membrane permeability, higher division rate, and structural changes in the cell wall of *A. baumannii* isolates, it could be proposed that during prolonged infections, persistent Cst^R^ can lead to reduced fitness, and lower *A. baumannii* virulence over time. However, our findings suggest that the opposite may occur, where enhanced *A. baumannii* virulence can be of greater benefit to some highly resistant local variants of MDR-AB. While the present study focused on the expression profile of four virulence genes among five Cst-R-AB isolates, the variation in the levels of virulence gene mRNA in vitro vs. in vivo conditions among Cst-R-AB isolates could be due to a myriad of additional factors, such as differences between the cellular environment of the wound site as compared to the cell-free culture conditions. Moreover, the role of tissue specific factors that dictate gene regulation, non-specific immune responses, remains to be investigated.

To our knowledge, this is the first report that attempts to establish an association between Cst^R^ among *A. baumannii* isolates and changes in the virulence genes expression. Our findings show the merits of a comprehensive investigation using a larger number of Cst-R-AB isolates from various regions of the world. Future detailed studies will shed light on the causal relationship between expression of a wider array of *A. baumannii* virulence genes, and genes that confer resistance to colistin, as well as other antimicrobial agents.

Finally, our findings highlight the intricate relationship between resistance to colistin, and the expression of *A. baumannii* virulence genes. It is commonly supposed that an increase in antimicrobial resistance leads, directly or indirectly, to reduced virulence of *A. baumannii* isolates. However, our data suggest that the opposite may occur, and it provides evidence that thorough investigations are warranted to directly measure the correlation between Cst^R^, reduced virulence, and “fitness” of *A. baumannii* isolates. Virulence gene expression variations among Cst-R-AB isolates might gain further clinical importance in health care settings, where isolates often transfer from patient to patient. Moreover, that the virulence of Cst-R-AB isolates may depend on their in vitro or in vivo conditions can help explain, at least in part, commonly encountered discrepancies between in vivo and in vitro laboratory test results. For instance, the recurrent failure of therapeutic antimicrobial agents in eradicating *A. baumannii* in patients’ burn wounds, despite the in vitro susceptibility of isolated pathogen to the same agents.

## Conclusions

This study concludes that the development of resistance to colistin among *A. baumannii* may be associated with virulence gene expression, which fluctuates under in vivo and in vitro conditions. We also provide evidence to speculate that enhanced virulence may be of greater benefit to highly-resistant variants of MDR-AB, and may give rise to formidable pathogens that increase morbidity among burn patients, and threaten the public health systems worldwide. Our findings warrant detailed examination of the interactions between virulence and antimicrobial resistance toward efforts to control the spread of multidrug-resistant *A. baumannii* (MDR-AB) isolates, and also to reduce disease severity in burn patients with MDR-AB infection.

## Additional file

**Additional file 1: Figure S1.** Comparison of relative change in the mRNA levels of virulence determinant genes *dnaK, blsA, recA,* and *lpsB* in cultures of a single Cst-R *A. baumannii* isolate, under in vitro and in vivo conditions. Bar indicates Mean + SD; five mice in each group. ^#^P < 0.01; *P < 0.05; ***P < 0.001.

## Abbreviations

AB: *Acinetobacter baumannii* or *A. baumannii*
MDR-AB: multidrug-resistant *A. baumannii*
Cst-R-AB: colistin-resistant *A. baumannii*
*recA*: gene encoding the RecA protein
*lpsB*: gene encoding the lipopolysaccharide core biosynthesis mannosyltransferase protein LpsB
*dnaK*: gene encoding the chaperone protein DnaK
*blsA*: gene encoding the BlsA (blue-light-sensing) photoreceptor protein
MDR: multi-drug resistant
XDR: extensive-drug resistant
GmbH: Gesellschaft mit beschränkter Haftung, which means “company with limited liability”
Cst^R^: colistin resistance
TUMS: Tehran University of Medical Sciences
*gyrB*: gene encoding the GyrB subunit of DNA gyrase protein
PCR: polymerase chain reaction
MHB: Mueller–Hinton Broth
DAD: disk agar diffusion
CLSI: Clinical and Laboratory Standards Institute
MIC: minimum inhibitory concentration
EUCAST: European Committee on Antimicrobial Susceptibility Testing
LPS: lipopolysaccharide
C-Burn: Mock-infected burned mice that serve as controls
*ompA*: gene encoding outer membrane protein A
*bla*_OXA-51_: the gene encoding the carbapenemase OXA-51
ompA: outer membrane protein A
csuE: chaperone-subunit usher E
MLVA: multi-loci variable-number tandem repeat analysis
DNA: deoxyribonucleic acid
ng: nanogram
µL: microliter
mM: millimolar
MgCl_2_: magnesium chloride
U: units
Taq: *Thermus aquaticus*
μM: micromolar
MIQE: minimum information for publication of quantitative real-time PCR experiments
dNTP: deoxyribonucleotide triphosphate
et al.: Et alii meaning “and other people”
s: second(s)
cm: centimeter(s)
%: percent
h: hour(s)
V: volt(s)
X: times
TBE: tris–borate-EDTA
bp: base pair(s)
VNTRs: variable number tandem repeats
L-VNTRs: large variable number tandem repeats
S-VNTRs: small variable number tandem repeats
μg: microgram(s)
v.: version
BHI: brain–heart-infusion
LB: Luria–Bertani
CFU: colony forming unit(s)
dH_2_O: distilled water
RT: room temperature
A: optical absorbance
Nm: nanometer(s)
PDR: pan-drug resistant
ATCC: American Type Culture Collection
NC: negative control
N: negative isolate
M: moderately positive isolate
S: strongly positive isolate
A_I_: initial absorbance
A_NC_: absorbance of negative control
^®^: registered trademark
wk: week
g: gram
No.: number
IC: international clone
i.p.: intraperitoneal
mm: millimeter
kg: kilogram
RNA: ribonucleic acid
cDNA: complementary DNA
PBS: phosphate-buffered saline
N_2_: nitrogen
S: svedberg unit(s) (e.g. 16S)
rRNA: ribosomal ribonucleic acid
mRNA: mitochondrial ribonucleic acid
USA: United States of America
RT-PCR: reverse transcription PCR
qRT-PCR: quantitative real-time RT-PCR
DNase I: deoxyribonuclease I
°C: degrees centigrade
pmol: picomolar
ng: nanogram(s)
min: minute(s)
ddH_2_O: double distilled water
REST: Relative Expression Software Tool
>: greater than
*n*-fold difference: Relative fold difference as compared to the basal gene expression
MT: MLVA type(s)
W-biofilm formers: weak biofilm formers
M-biofilm formers: moderate biofilm formers
S-biofilm formers: strong biofilm formers
Suppl.: supplement
SD: standard deviation
