# Supplementary figures and images for: Association of virulence gene expression with colistin-resistance in Acinetobacter baumannii: analysis of genotype, antimicrobial susceptibility, and biofilm formation
Source: Ann Clin Microbiol Antimicrob. 2018 Jun 1;17:24. doi: 10.1186/s12941-018-0277-6 (PMC5984448; doi:10.1186/s12941-018-0277-6)

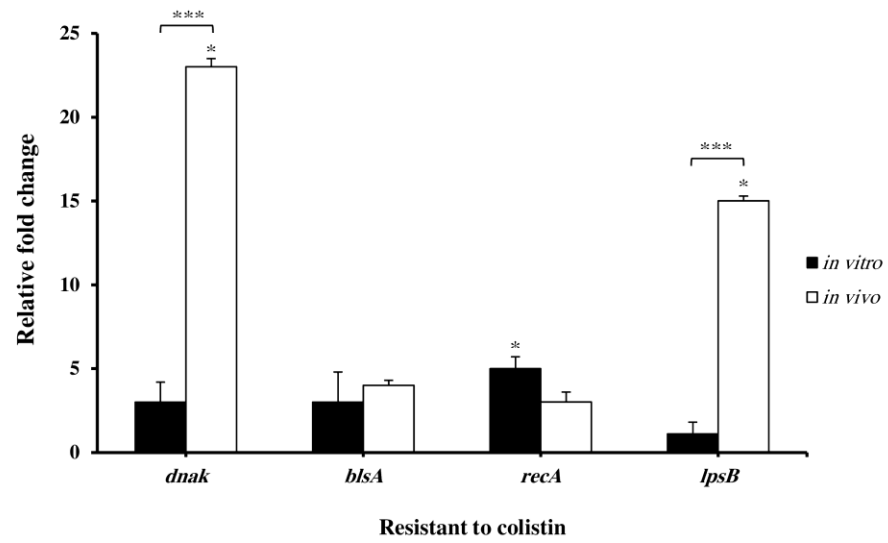

Supplement: Supplementary file 1 — Additional file 1: Figure S1. Comparison of relative change in the mRNA levels of virulence determinant genes dnaK, blsA, recA, and lpsB in cultures of a single Cst-R A. baumannii isolate, under in vitro and in vivo conditions. Bar indicates Mean + SD; five mice in each group. #P < 0.01; *P < 0.05; ***P < 0.001. [file 12941_2018_277_MOESM1_ESM.pdf]
